# YouTube/ Bilibili/ TikTok videos as sources of medical information on laryngeal carcinoma: cross-sectional content analysis study

**DOI:** 10.1186/s12889-024-19077-6

**Published:** 2024-06-14

**Authors:** ZeYang Liu, YiWen Chen, Ying Lin, MaoMao Ai, DongLing Lian, YuanHui Zhang, YanXiong Shen, Feng Yu

**Affiliations:** Institute of Otorhinolaryngology-Head and Neck Surgery, Guangzhou Red Cross Hospital of Jinan University, Guangzhou, China

**Keywords:** Laryngeal cancer, Social media, Patient education, Public education, Public health, Information quality, YouTube, TikTok, Bilibili

## Abstract

**Background:**

YouTube, a widely recognized global video platform, is inaccessible in China, whereas Bilibili and TikTok are popular platforms for long and short videos, respectively. There are many videos related to laryngeal carcinoma on these platforms. This study aims to identify upload sources, contents, and feature information of these videos on YouTube, Bilibili, and TikTok, and further evaluate the video quality.

**Methods:**

On January 1, 2024, we searched the top 100 videos by default sort order (300 videos in total) with the terms “laryngeal carcinoma” and “throat cancer” on YouTube, “喉癌” on Bilibili and TikTok. Videos were screened for relevance and similarity. Video characteristics were documented, and quality was assessed by using the Patient Education Materials Assessment Tool (PEMAT), Video Information and Quality Index (VIQI), Global Quality Score (GQS), and modified DISCERN (mDISCERN).

**Results:**

The analysis included 99 YouTube videos, 76 from Bilibili, and 73 from TikTok. Median video lengths were 193 s (YouTube), 136 s (Bilibili), and 42 s (TikTok). TikTok videos demonstrated higher audience interaction. Bilibili had the lowest ratio of original contents (69.7%). Treatment was the most popular topic on YouTube and Bilibili, while that was the prognosis on TikTok. Solo narration was the most common video style across all platforms. Video uploaders were predominantly non-profit organizations (YouTube), self-media (Bilibili), and doctors (TikTok), with TikTok authors having the highest certification rate (83.3%). Video quality, assessed using PEMAT, VIQI, GQS, and mDISCERN, varied across platforms, with YouTube generally showing the highest scores. Videos from professional authors performed better than videos from non-professionals based on the GQS and mDISCERN scores. Spearman correlation analysis showed no strong relationships between the video quality and the audience interaction.

**Conclusions:**

Videos on social media platforms can help the public learn about the knowledge of laryngeal cancer to some extent. TikTok achieves the best flow, but videos on YouTube are of the best quality. However, the video quality across all platforms still needs enhancement. We need more professional uploaders to ameliorate the video quality related to laryngeal carcinoma. Content creators also should be aware of the certification, the originality, and the style of video shooting. As for the platforms, refining the algorithm will allow users to receive more high-quality videos.

**Supplementary Information:**

The online version contains supplementary material available at 10.1186/s12889-024-19077-6.

## Introduction

Many Internet users use online video platforms for medical information [1]. The internet has shifted patients’ roles from passive information recipients to active information searchers, thereby increasing patient activation in managing their health care [2]. However, inaccurate information may mislead patients to make wrong decisions and even influence their outcomes [3, 4]. Therefore, enhancing the quality and content of these health-related online videos can improve the public’s accurate perception of health [5].

YouTube, recognized as the most extensive global long-video platform [6, 7], remains inaccessible in China. Bilibili and TikTok, which dominate the long-video and short-video segments in the Chinese market respectively have filled this gap [5, 8, 9].

Although laryngeal cancer accounts for only 1% of total cancer cases and related deaths, it is one of the most prevalent types of head and neck cancer [10]. There are many videos related to laryngeal carcinoma on these platforms. Nevertheless, the quality evaluation of these videos remains sparse [11]. This study aims to identify upload sources, contents, and feature information of these videos on YouTube/ Bilibili/ TikTok and further evaluate the video quality. We expect to provide suitable directions for the public to learn about laryngeal carcinoma from online videos and give comprehensive advice to content creators and platforms.

## Materials and methods

### Ethical considerations

All information was obtained from publicly released YouTube, Bilibili, and TikTok videos, and none of the data involved personal privacy concerns. Therefore, no ethics review was needed.

### Video collection

On January 1, 2024, a search was performed on YouTube with the keywords “laryngeal carcinoma” and “throat cancer” in English. Bilibili and TikTok were searched using the term “喉癌” (laryngeal carcinoma in Chinese. This is both a scientific name and a common colloquial term and the same in simplified Chinese and traditional Chinese characters.). Before searching, we logged out of all accounts and cleared the search history to avoid bias from personalized recommendations. The search results were presented in the default order without any filtering criteria. We skipped the videos published within a week because the data on views and likes were unstable and could not accurately reflect audience engagement. Advertising videos were also skipped (Additional file 1). The top 100 videos on each platform were collected.

### Video characteristics

On January 1, 2024, various attributes of the videos were systematically documented, including video length, duration, views, views/30 days, thumbs up, thumbs up/30 days, comments, comments/30 days, coins, collections, collections/30 days, shares, and shares/30 days. However, the following data were unavailable: ① views on TikTok, ② collections and shares on YouTube.

### Uploader characteristics

Similarly, on the same day, details regarding the uploaders were gathered, including ID, number of fans, certification status, and type. Certification was determined based on specific criteria (Additional file 1). The video uploaders were categorized as doctors, other medical workers/students, hospitals/ departments/ associations (also regarded as non-profit organizations), for-profit companies, official media (the media under government regulation, such as BBC), and self-media. Self-media was regarded as non-professionals, others as professionals.

### Video review and categorization

Between January 2 and 7, 2024, two authors (ZY.L. and YW.C.) independently reviewed the videos and excluded some similar or irrelevant videos (Additional file 1). The topic of the videos was categorized as anatomy, etiology/ prevention, pathology, epidemiology, symptoms, examinations/ diagnosis, treatment, and prognosis. The number of topics covered by each video was collected, as some videos were related to multiple topics. Videos not covering these topics were deemed irrelevant and should be excluded.

### Originality and style assessment

Videos that were direct reprints, translations, or gross editions were not considered original (Additional file 1). The style of video shooting was classified as solo narration, questions and answers (Q&A), PPT/class, animation/action, medical scenarios, TV show/documentary, and others (Additional file 1).

### Quality assessment

Two authors (ZY.L. and YW.C.) independently assessed the quality of the remaining videos from January 8 to 18, 2024. A third arbitrator (Y.L.) assigned the final score if the two raters’ scores were inconsistent. Furthermore, we used Cohen’s kappa (κ) to quantify the agreement between the two raters. The Patient Education Materials Assessment Tool (PEMAT), Video Information and Quality Index (VIQI), Global Quality Score (GQS), and modified DISCERN (mDISCERN) were utilized to evaluate the video quality.

The PEMAT [12] consists of 25 questions, with 21 representing the understandability of health information and 4 evaluating the actionability of recommendations by videos. Each item is scored as “agree = 1, disagree = 0, N/A”. The total score (PEMAT-T) and the score of understandability (PEMAT-U) and actionability section (PEMAT-A) are calculated as “Total Points/Total Possible Points × 100”. Higher scores indicate better performance.

The VIQI tool [13] encompasses four dimensions: information flow (VIQI 1), information accuracy (VIQI 2), quality (videos including one point for each image, animation, interview, video captions, and summary) (VIQI 3), and precision (level of coherence between video title and content) (VIQI 4). Each criterion is rated on a scale of 1 to 5, with higher scores indicating better quality.

The GQS [14], a 5-point scale, assessed overall video quality, ranging from poor (1) to excellent (5).

The mDISCERN was adapted from the DISCERN tool and is more suitable for assessing video material [6, 15]. It consists of five questions related to the reliability of the video. Each question is scored 1 for “yes” and 0 for “no”. Higher scores correspond to more excellent reliability.

Previous studies have validated the above tools, particularly in the context of social media platforms [5–9, 12–14]. The Additional file 2 and the Additional file 3 provide detailed descriptions of these tools.

### Statistical analysis

We used IBM SPSS version 24.0 to analyze the data. Shapiro–Wilk test was applied for testing the normality of continuous variables. Normally distributed continuous variables were presented as mean ± SD (standard deviation), while those without normal distribution were presented as median, min–max values, and 25–75 percentiles (M[P25, P75]). We used Cohen κ to quantify the agreement between the two raters. The κ values were interpreted as follows: κ > 0.8 indicated excellent consistency; 0.6 < κ ≤ 0.8 suggested substantial agreement; 0.4 < κ ≤ 0.6 signified moderate agreement; and κ ≤ 0.4 was indicative of poor agreement. The Mann–Whitney U test was applied to compare continuous variables without normal distribution. Categorical variables were reported as numbers and rates. The Chi-square test, continuity correction, or Fisher’s exact test were utilized for comparing categorical variables. A pairwise comparison was employed to elucidate differences among the three platforms. We performed Spearman correlation analysis to evaluate the relationship between audience interaction and video quality. Spearman’s rank correlation coefficient (r) was used, with r > 0 denoting a positive correlation and r < 0 indicating a negative correlation. The strength of the correlation was classified as follows: |r|≤ 0.2 represented no relationship; 0.2 <|r|≤ 0.4 implied a weak relationship; 0.4 <|r|≤ 0.6 indicated a moderate relationship; 0.6 <|r|≤ 0.8 suggested a strong relationship; and |r|> 0.8 denoted a very strong relationship. A value of *P* < 0.05 was considered statistically significant.

## Results

### Video characteristics

**Fig. 1:**
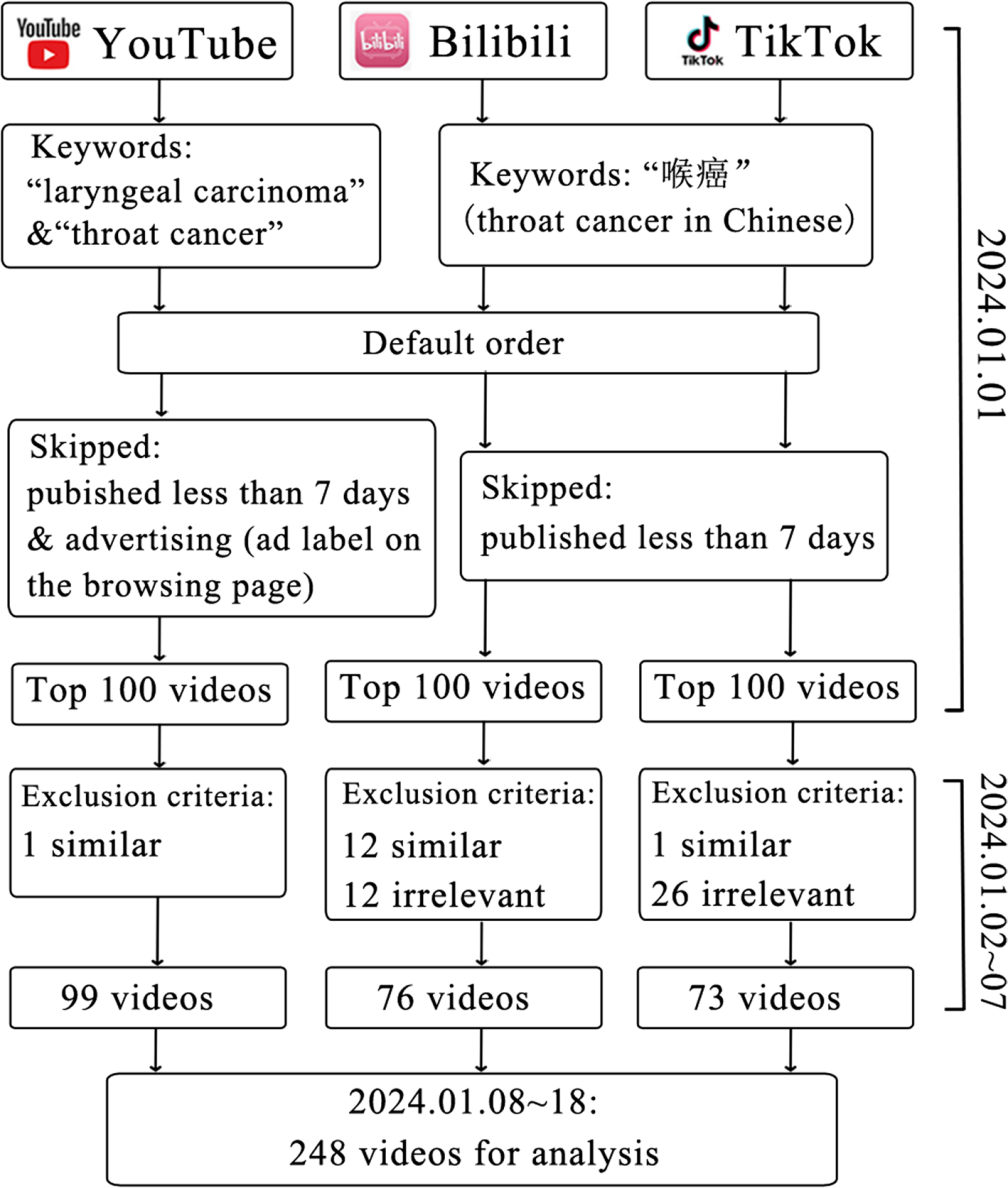
Search strategy for videos on laryngeal cancer

Our study included 99 YouTube videos, 76 from Bilibili and 73 from TikTok, after excluding duplicates and non-relevant content (Fig. 1). The irrelevant content included celebrities with laryngeal cancer and patients seeking sympathy. All videos on YouTube were in English or had English subtitles. Similarly, all videos on Bilibili and TikTok were in Chinese or had Chinese subtitles. The characteristics of the videos from YouTube, Bilibili, and TikTok are detailed in Table 1. All the continuous variables were not normally distributed according to the Shapiro–Wilk test. TikTok videos (42[28.5–74] seconds) were notably shorter compared to those on YouTube (193[102–467] seconds) and Bilibili (136[89.25–276] seconds), and were generally newer based on their upload dates. TikTok led in audience interaction, showing the highest thumbs up and comments, while Bilibili exhibited the least interaction across all metrics.

**Table 1:** Characteristics of videos about laryngeal carcinoma on YouTube/ Bilibili/ TikTok

Platform	YouTube(N_1_ = 99)			Bilibili(N_2_ = 76)			TikTok(N_3_ = 73)			*P*-value		
Characteristic	M	Min–Max	P25-P75	M	Min–Max	P25-P75	M	Min–Max	P25-P75	P_Y-B_	P_B-T_	P_Y-T_
Video length(s)	193	6–7438	102–467	136	31–8986	89.25–276	42	6–2575	28.5–74	0.194	** < 0.001**	** < 0.001**
Duration(day)	1365	72–5655	661–2492	547.5	11–2719	260.5–962.25	331	18–1355	209–682	** < 0.001**	**0.048**	** < 0.001**
Views	9909	238–1530594	2852–35422	655	72–1266000	250–2005.25	-	-	-	** < 0.001**	-	-
Views/30 days	243.6	11.0–43286.6	95.4–839.0	47.4	1.68–60381.6	18.25–136.5	-	-	-	** < 0.001**	-	-
Thumbs up^a^	71	2–6437	28–294	8	0–64000	3.25–23.75	547	8–112000	206.5–1304.5	** < 0.001**	** < 0.001**	** < 0.001**
Thumbs up^a^ /30 days	1.92	0.04–139.4	0.61–7.73	0.58	0–3052.5	0.167–1.38	57.0	0.41–22105.3	21.3–152.4	** < 0.001**	** < 0.001**	** < 0.001**
Comments^b^	6	0–694	0.5–28.5	0	0–441	0–3	41	0–4591	14.5–95	** < 0.001**	** < 0.001**	** < 0.001**
Comments^b^ /30 days	0.17	0–12.7	0.00–0.72	0.00	0–21.0	0.00–0.25	4.33	0–547.1	1.34–10.4	** < 0.001**	** < 0.001**	** < 0.001**
Collections	-	-	-	5	0–4365	2–24.75	68	0–3645	37–182.5	-	** < 0.001**	-
Collections /30 days	-	-	-	0.36	0–208.2	0.10–1.72	9.37	0–719.4	2.58–21.5	-	** < 0.001**	-
Shares	-	-	-	4	0–413	1–13	116	0–7259	40.5–327	-	** < 0.001**	-
Shares/30 days	-	-	-	0.19	0–25.6	0.07–0.91	8.13	0–631.2	3.85–25.2	-	** < 0.001**	-

All the *P*-values were obtained from Mann–Whitney U test

P_Y-B_: YouTube versus Bilibili; P_B-T_: Bilibili versus TikTok; P_Y-T_: YouTube versus TikTok. Bold text means the *P*-value < 0.05

^a^Excluded: 2 videos on YouTube turned off the function of thumbs-up

^b^Excluded: 14 videos on YouTube turned off the function of comments

### Uploader characteristics

**Fig. 2:**
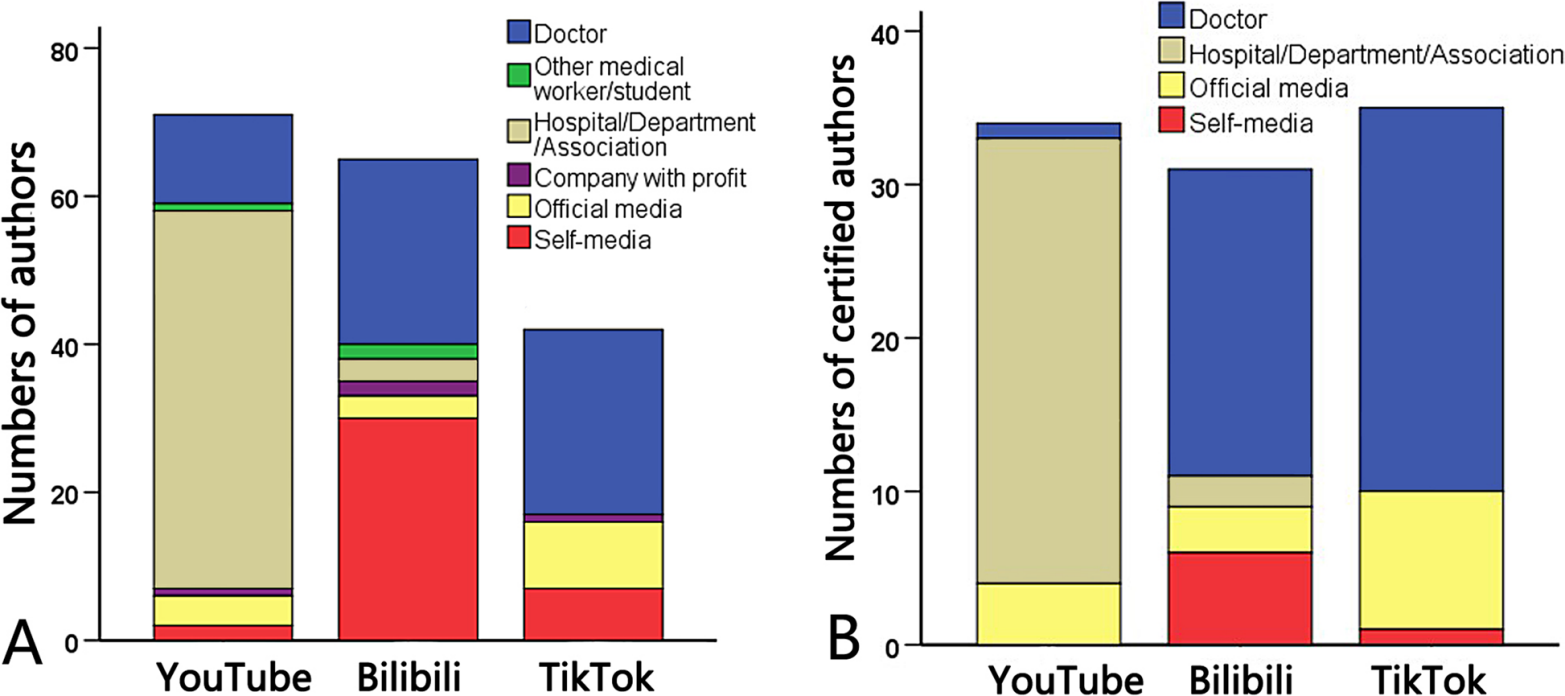
Numbers of video uploaders about laryngeal carcinoma on YouTube/ Bilibili/ TikTok. **A** All the authors. **B** the Certified authors

In this study, there were 71 uploaders on YouTube, 65 on Bilibili, and 42 on TikTok (Table 2). TikTok authors owned the largest number of followers and uploaded videos more frequently, contrasting with the lower activity among Bilibili authors. The categories of uploaders varied significantly across platforms (Fig. 2A). Nearly three-quarters of the authors on YouTube were from non-profit organizations (hospitals/departments/associations), followed by individual doctors. Bilibili uploaders mainly consisted of self-media, with doctors as the second-largest group. On TikTok, over half of the accounts belonged to doctors, followed by official media. TikTok uploaders had the highest rate of certification (83.3%), with all doctors being certified (Fig. 2B). In addition, seven uploaders on Bilibili were doctors of traditional Chinese medicine (TCM), six of whom were certified.

**Table 2:** Characteristics of video uploaders about laryngeal carcinoma on YouTube/ Bilibili/ TikTok

Platform	YouTube	Bilibili	TikTok	P_Y-B_	P_B-T_	P_Y-T_
Number of uploaders	71	65	42	-	-	-
Followers, Median[P25,P75]	29000[9840,171000]	1917[242.5,15241.5]	58000[14500,440750]	** < 0.001**^**a**^	** < 0.001**^**a**^	0.308^**a**^
Number of videos per person, Mean ± SD, Median[P25,P75]	1.39 ± 1.05, 1[1,1]	1.17 ± 0.45, 1[1,1]	1.74 ± 1.59, 1[1, 2]	0.393^**a**^	0.068^**a**^	0.276^**a**^
Type of uploaders, n(%)						
Doctor	12(16.9%)	25(38.5%)	25(59.5%)	**0.005**^**b**^	**0.033**^**b**^	** < 0.001**^**b**^
Other medical worker/student	1(1.4%)	2(3.1%)	0	0.938^c^	0.519^d^	1.000^d^
Non-profit organization	51(71.8%)	3(4.6%)	0	** < 0.001**^**b**^	0.416^c^	** < 0.001**^**b**^
Company with profit	1(1.4%)	2(3.1%)	1(2.4%)	0.938^c^	1.000^c^	1.000^d^
Official media	4(5.6%)	3(4.6%)	9(21.4%)	1.000^c^	**0.017**^**c**^	**0.025**^**c**^
Self-media	2(2.9%)	30(46.2%)	7(16.7%)	** < 0.001**^**b**^	**0.002**^**b**^	**0.023**^**c**^
Doctor of TCM, n(%)	0(0%)	7(10.8%)	0(0%)	**0.014**^**c**^	0.072^c^	-
Certification, n(%)	34(47.9%)	31(47.7%)	35(83.3%)	0.982^**b**^	** < 0.001**^**b**^	** < 0.001**^**b**^
Type of certified uploaders, n(%)						
Doctor	1(2.9%)	20(64.5%)	25(71.4%)	** < 0.001**^**b**^	0.547^**b**^	** < 0.001**^**b**^
Other medical worker/student	0	0	0	-	-	-
Non-profit organization	29(85.3%)	2(6.5%)	0	** < 0.001**^**b**^	0.217^d^	** < 0.001**^**b**^
Company with profit	0	0	0	-	-	-
Official media	4(11.8%)	3(9.7%)	9(25.7%)	1.000^c^	0.092^**b**^	0.138^**b**^
Self-media	0	6(19.4%)	1(2.9%)	**0.024**^**c**^	0.076^c^	1.000^d^

P_Y-B_: YouTube versus Bilibili; P_B-T_: Bilibili versus TikTok; P_Y-T_: YouTube versus TikTok. Bold text means the *P*-value < 0.05

^a^Mann-Whitney U test

^b^Chi-squared test

^c^Continuity correction

^d^Fisher’s exact test

### Video categorization

Video categorization is shown in Table 3. Original content was predominant on YouTube (98.0%) and TikTok (94.5%) but less on Bilibil(69.7%). Topic variety differed across platforms, with YouTube and Bilibili videos covering more topics than TikTok, attributed to their longer duration. Treatment and symptoms were the most and the second most popular topics on YouTube and Bilibili. Many videos addressed the different symptoms of laryngeal cancer and chronic pharyngitis. Transoral laser microsurgery, an ideal treatment for early-stage laryngeal cancer [16], was frequently mentioned in several videos. Prognosis topped the topic on TikTok, followed by treatment, mainly due to patients sharing their postoperative experiences (especially about how to talk after surgery). Some doctors also liked shooting postoperative patient follow-up visits in the outpatient department.

**Table 3:** Categorization of videos about laryngeal carcinoma on YouTube/ Bilibili/ TikTok

Platform	YouTube(N1 = 99)	Bilibili(N2 = 76)	TikTok(N3 = 73)	P_Y-B_	P_B-T_	P_Y-T_
Originality, n(%)	97(98.0%)	53(69.7%)	69(94.5%)	** < 0.001**^**b**^	** < 0.001**^**b**^	0.423^c^
Number of topics per video, Median[P25,P75]	2[1, 3]	1[1, 2]	1[1]	0.062^a^	**0.003**^**a**^	** < 0.001**^**a**^
Type of topics, n(%)						
Anatomy	19(19.2%)	18(23.7%)	1(1.4%)	0.471^b^	** < 0.001**^**b**^	** < 0.001**^**b**^
Etiology/Prevention	23(23.2%)	25(32.9%)	10(13.7%)	0.156^b^	**0.006**^**b**^	0.117^b^
Pathology	10(10.1%)	8(10.5%)	1(1.4%)	0.927^b^	**0.045**^**c**^	0.046^c^
Epidemiology	13(13.1%)	7(9.2%)	1(1.4%)	0.419^b^	0.079^c^	**0.005**^**b**^
Symptoms	50(50.5%)	29(38.2%)	16(21.9%)	0.104^b^	**0.031**^**b**^	** < 0.001**^**b**^
Examinations/Diagnosis	38(38.4%)	17(22.4%)	11(15.1%)	**0.024**^**b**^	0.254^b^	**0.001**^**b**^
Treatment	58(58.6%)	33(43.4%)	20(27.4%)	0.047^b^	**0.041**^**b**^	** < 0.001**^**b**^
Prognosis	24(24.2%)	15(19.7%)	37(50.7%)	0.478^b^	** < 0.001**^**b**^	** < 0.001**^**b**^
TCM, n(%)	0(0%)	21(27.6%)	1(1.4%)	** < 0.001**^**b**^	** < 0.001**^**b**^	0.424^d^
Style of video shooting, n(%)						
Solo narration	25(25.5%)	25(32.9%)	29(39.7%)	0.267^b^	0.386^b^	**0.043**^**b**^
Question & Answer	10(10.1%)	5(6.6%)	2(2.8%)	0.409^b^	0.472^c^	0.061^**b**^
PPT or Class	22(22.2%)	14(18.4%)	0	0.538^b^	** < 0.001**^**b**^	** < 0.001**^**b**^
Animation / Action	17(17.2%)	6(7.9%)	3(4.1%)	0.072^b^	0.532^c^	**0.008**^**b**^
Medical scenarios	4(4.0%)	8(10.5%)	10(13.9%)	0.092^b^	0.553^b^	**0.022**^**b**^
TV show / Documentary	5(5.1%)	3(3.9%)	6(8.2%)	1.000^c^	0.453^c^	0.600^c^
Others	16(16.3%)	15(19.7%)	23(31.5%)	0.539^b^	0.099^b^	**0.018**^**b**^

P_Y-B_: YouTube versus Bilibili; P_B-T_: Bilibili versus TikTok; P_Y-T_: YouTube versus TikTok. Bold text means the *P*-value < 0.05

^a^Mann-Whitney U test

^b^Chi-squared test

^c^Continuity correction

^d^Fisher’s exact test

The style of video shooting also varied, with solo narration being the most common across platforms and PPT/class more prevalent in long-video platforms.

Additionally, 21 videos on Bilibili focused on TCM, but only 12 were from professional uploaders. Only one video on TikTok mentioned TCM, uploaded by a self-media account.

### Video quality

The κ value indicating interobserver reliability was 0.78. Overall, YouTube videos were of the best quality because it was statistically significant that they had the highest scores in the VIQI-sum, GQS, and mDISCERN-sum (Table 4). Despite similar PEMAT-T scores across all the platforms, TikTok videos scored highest in PEMAT-U and lowest in PEMAT-A. Approximately, there was little difference between the scores of Bilibili and TikTok. Professional authors generally outperformed non-professionals in GQS and mDISCERN, according to Table 5.

**Table 4:** Quality assessment of videos about laryngeal carcinoma on YouTube/ Bilibili/ TikTok

Platform	YouTube(N1 = 99)			Bilibili(N2 = 76)			TikTok(N3 = 73)			*P*-value		
Scores	M	Min–Max	P25-P75	M	Min–Max	P25-P75	M	Min–Max	P25-P75	P_Y-B_	P_B-T_	P_Y-T_
PEMAT-T	71.4	33.3–100.0	66.7–84.7	76.9	42.9–100.0	69.2–89.2	75.0	44.4–100.0	69.2–90.0	0.245^a^	0.915^a^	0.215^a^
PEMAT-U	72.7	50.0–100.0	66.7–88.9	72.7	40.0–100.0	65.2–88.5	85.7	50.0–100.0	70.0–100.0	0.337^a^	**0.001**^a^	** < 0.001**^a^
PEMAT-A	66.7	0.0–100.0	66.7–100.0	66.7	0.0–100.0	66.7–100.0	66.7	0.0–100.0	33.3–66.7	0.532^a^	** < 0.001**^a^	** < 0.001**^a^
VIQI-sum	15	10–19	13–17	12	8–18	11–15	13	8–17	12–14	** < 0.001**^a^	0.359^a^	** < 0.001**^a^
VIQI-1	2	1–4	2–3	1	1–5	1–2	3	1–5	3–4	** < 0.001**^**a**^	** < 0.001**^a^	** < 0.001**^a^
VIQI-2	5	2–5	4–5	4	2–5	4–5	4	2–5	3–4.5	** < 0.001**^a^	0.079^a^	** < 0.001**^a^
VIQI-3	3	1–5	2–5	2	1–5	1–4	1	1–5	1–2	**0.035**^a^	**0.002**^**a**^	** < 0.001**^a^
VIQI-4	5	3–5	5–5	5	2–5	5–5	5	1–5	4–5	0.101^a^	0.294^a^	**0.007**^a^
GQS	4	2–5	3–4	3	2–4	3–4	3	2–4	2.5–4	** < 0.001**^a^	0.791^a^	** < 0.001**^a^
mDISCERN-sum	3	1–5	3–4	3	0–5	2–4	3	1–4	3–3	0.120^a^	0.077^a^	** < 0.001**^a^
mDISCERN-1	93(93.9%)			69(90.8%)			67(91.8%)			0.431^b^	0.830^b^	0.583^b^
mDISCERN-2	98(99.0%)			65(85.5%)			61(83.6%)			** < 0.001**^b^	0.740^b^	** < 0.001**^b^
mDISCERN-3	95(96.0%)			55(72.4%)			67(91.8%)			** < 0.001**^b^	**0.002**^b^	0.408^c^
mDISCERN-4	16(16.2%)			11(14.5%)			1(1.4%)			0.759^b^	**0.003**^b^	**0.001**^b^
mDISCERN-5	32(32.3%)			30(39.5%)			7(9.6%)			0.327^b^	** < 0.001**^b^	** < 0.001**^b^

P_Y-B_: YouTube versus Bilibili; P_B-T_: Bilibili versus TikTok; P_Y-T_: YouTube versus TikTok. Bold text means the *P*-value < 0.05

^a^Mann-Whitney U test

^b^Chi-squared test

^c^Continuity correction

**Table 5:** Quality comparison between the videos uploaded by professionals and non-professionals

Scores	Professionals (*N* = 200)			Non-professionals (*N* = 48)			*P*-value
	M	Min–Max	P25-P75	M	Min–Max	P25-P75	
PEMAT-T	76.0	33.3–100.0	69.2–90.0	71.4	44.4–100.0	62.2–78.6	0.080^a^
PEMAT-U	72.7	50.0–100.0	66.7–100.0	72.7	40.0–100.0	60.9–83.3	0.080^a^
PEMAT-A	66.7	0.0–100.0	66.7–100.0	66.7	0.0–100.0	66.7–100.0	0.979^a^
VIQI-sum	14	8–19	12–16	13	8–18	12–15	0.783^a^
VIQI-1	2	1–5	2–3	2	1–5	1–3	**0.049**^a^
VIQI-2	4	2–5	4–5	4	2–5	3–4.75	** < 0.001**^a^
VIQI-3	2	1–5	1–4	2	1–5	1–4	0.158^a^
VIQI-4	5	1–5	5–5	5	1–5	4–5	0.242^a^
GQS	3	2–5	3–4	3	2–4	2–4	**0.002**^a^
mDISCERN-sum	3	0–5	3–4	3	0–5	2–3.75	**0.006**^a^
mDISCERN-1	185(92.5%)			44(91.7%)			1.000^c^
mDISCERN-2	193(96.5%)			31(64.6%)			** < 0.001**^**c**^
mDISCERN-3	181(90.5%)			36(75.0%)			**0.004**^**b**^
mDISCERN-4	21(10.5%)			7(14.6%)			0.422^b^
mDISCERN-5	55(27.5%)			14(29.2%)			0.817^b^

Professionals: doctor, other medical worker/student, hospital/department/association, medical company with profit, and official media

Non-professionals: self-media

^a^Mann-Whitney U test

^b^Chi-squared test

^c^Continuity correction

### Correlation analysis

No strong relationships were found between the video quality and the audience interaction (Table 6). Approximately, VIQI, GQS, and mDISCERN had weak to moderate positive relationships with the audience interaction. PEMAT scores appeared mostly unrelated to audience interaction. Unexpectedly, on TikTok, mDISCERN negatively correlated to thumbs-up(moderate), comments(weak), and shares(weak).

**Table 6:** Spearman correlation between video quality and audience interaction on YouTube/ Bilibili/ TikTok

r, *P*-value	YouTube (N1 = 99)				Bilibili (N2 = 76)				TikTok (N3 = 73)			
	PEMAT	VIQI	GQS	mDISCERN	PEMAT	VIQI	GQS	mDISCERN	PEMAT	VIQI	GQS	mDISCERN
Views	-0.092, 0.366	0.346, < **0.001**	0.250, **0.012**	-0.089, 0.379	-0.184, 0.112	0.415, < **0.001**	0.356, **0.002**	0.191, 0.099	-	-	-	-
Thumbs-up^a^	0.027, 0.795	0.449, < **0.001**	0.350 < **0.001**	0.055, 0.592	-0.044, 0.705	0.489, < **0.001**	0.387, **0.001**	0.255, **0.026**	-0.206, 0.081	0.271, **0.021**	-0.095, 0.423	-0.435, < **0.001**
Comments^b^	-0.026, 0.816	0.299, **0.005**	0.236, **0.029**	-0.009, 0.933	-0.216, 0.060	0.112, 0.334	0.142, 0.221	0.039, 0.737	-0.253, **0.031**	0.176, 0.136	-0.160, 0.176	-0.378, **0.001**
Collections	-	-	-	-	0.024, 0.838	0.549, < **0.001**	0.449, < **0.001**	0.252, **0.028**	-0.005, 0.966	0.389, **0.001**	0.158, 0.182	-0.116, 0.330
Shares	-	-	-	-	-0.037, 0.751	0.508, < **0.001**	0.412, < **0.001**	0.305, **0.007**	-0.162, 0.171	0.337, **0.004**	0.079, 0.505	-0.239, **0.041**

Bold text means the *P*-value < 0.05

|r|≤ 0.2 no relationship; 0.2 <|r|≤ 0.4 weak relationship; 0.4 <|r|≤ 0.6 moderate relationship; 0.6 <|r|≤ 0.8 strong relationship; |r|> 0.8 very strong relationship

^a^Excluded: 2 videos on YouTube turned off the function of thumbs-up

^b^Excluded: 14 videos on YouTube turned off the function of comments

## Discussion

The use of social media in public health education has been increasing due to its ability to remove physical barriers that traditionally impede access to healthcare support and resources [17–19]. In recent decades, digital video has been widely used as an important information carrier for patients’ education.

In the field of otorhinolaryngology head and neck surgery, YouTube and TikTok content has been investigated for the educational value of videos about cholesteatoma [20], pediatric tonsillectomy [21], middle ear ventilation tubes [22], rhinoplasty [23], tinnitus [24], nasopharyngeal carcinoma [25], and thyroid cancer [8, 26, 27]. However, the overall quality of these videos was not satisfying. Similar studies also raised concerns about the misinformation on social media and called for the responsibility of health specialists to improve health-related content [4, 19].

Few articles concerning laryngeal cancer-related videos have been published, except for Narwani’s research in 2016 [11]. However, his research only involved 54 videos from Google/ Yahoo/ Bing/ MSN and suggested much of the laryngeal cancer information was of suboptimal quality and written at a level too difficult for the average adult to read comfortably [11]. With the rapid growth of social media platforms, the findings may have varied recently.

### Principal findings

This study is the first comprehensive evaluation of laryngeal cancer-related videos across major video platforms. Research on Bilibili remains sparse, and we filled this gap. Our study also revealed the differences between YouTube, Bilibili, and TikTok through detailed statistical pairwork comparisons. We used four tools to make a comprehensive judgment that YouTube videos performed relatively the best but still needed improvement. We considered factors like originality, certification, video shooting style, and TCM—elements often overlooked in previous research. Our findings not only guide public access to health information but also provide helpful insights for both content creators and the platforms.

### Video characteristics

The difference in video platform histories and the difference in algorithms explain the variance in video duration and relevance, respectively. YouTube, established in 2005 [28], may have less priority of the publication date during searching (default order according to “relevance”), whereas Bilibili (established in 2009 [9]) and TikTok (established in 2016 [29]) use a complex algorithm incorporating recency and user engagement (default order according to “comprehensiveness” [9]).

Interestingly, despite lacking health information, some irrelevant videos on TikTok/Bilibili achieved high viewership and engagement. This phenomenon was also found in the studies on other diseases [30–32]. Those videos were most likely to contain hot topics (such as celebrities). Besides, videos from patients might arouse public compassion and achieve much flow. This suggests a potential strategy for video creators to increase audience interaction.

Despite being the newest platform, TikTok exhibited the highest engagement levels in our study. This aligns with studies suggesting that shorter videos tend to be more addictive and disseminate rapidly due to their suitability for consumption during brief intervals [33, 34]. This also explained why it had the highest score of VIQI-1 (information flow) and the largest number of followers.

These findings highlight the evolving landscape of online health information dissemination, where platform-specific characteristics significantly influence content relevance and audience engagement.

### Uploader characteristics

Our study reveals that TikTok authors tended to upload more videos, likely because longer videos can be segmented into shorter clips, inflating the number of uploads.

The diversity in uploader types across platforms could be attributed mainly to the varying certification policies of each platform. YouTube’s algorithm favors group accounts, making over half of its uploaders non-profit organizations. This finding on YouTube agrees with the previous studies on other diseases [5, 6, 20, 35]. TikTok, with its stringent certification requirements, only allows certified attending/associate/chief doctors from grade 3 and first-class hospitals (A hospital ranking system in China. Grade 3 and first-class means the top level) to use the title “doctor,” as corroborated by multiple studies [5, 8, 27, 36]. While enhancing content authenticity, this policy restricts the participation of resident doctors, grassroots doctors, and medical students in China, leading them to gravitate towards Bilibili, which has more relaxed certification processes. Meanwhile, Bilibili’s leniency in certification standards allows a greater number of non-professionals to publish health-related content, raising potential concerns about the quality and reliability of these videos. As creators will receive more support from the platforms after certification, we suggest all professionals apply for it.

It is said that China is now vigorously promoting TCM on several platforms, but few studies have mentioned TCM [8, 9]. According to the our research, Bilibili was aligned with Chinese health policy favoring TCM, but the videos posted by non-professionals raised quality and authenticity concerns. Zheng’s 2023 study on TikTok liver cancer videos noted lower-quality TCM content [9]. Unexpectedly, TikTok had only one TCM video. This contrasted sharply with Yang’s 2022 study, in which TCM videos comprised a quarter of TikTok’s top 100 thyroid cancer search results [8]. This discrepancy may be due to a recent strict rule of certification on TikTok (Additional file 1). Many TCM doctors who work in relatively lower-ranking hospitals are not allowed to post TCM videos on TikTok because they can not get certified. In conclusion, there is a clear need for improvements in the quality and representation of TCM content on these platforms.

### Video content

While previous studies have overlooked originality, it is crucial for content creators and platforms. Bilibili’s copyright policy (Additional file 1) mainly contributes to its lowest originality rate. Video uploaders should also be aware of copyright issues. Watermarks are recommended for original content. Uploaders should also avoid infringement when reposting or translating others’ works. All video platforms should protect and support authors who produce high-quality original content.

Longer videos on YouTube and Bilibili encompass a broader range of topics due to their capacity to convey more information. Consistent with our study, treatment and symptoms were the most common topics in some previous studies about other diseases [5, 6, 8]. Prognosis was the most popular topic on TikTok, mainly because short videos are more suitable for sharing life. The videos on TikTok about prognosis reflect that patients with laryngeal cancer can usually receive ideal treatment in China.

There are no studies examining the style of video shooting. Based on our experience, solo narration has always been the predominant video style across all platforms, likely due to its ease of production, particularly for individual creators like doctors with limited time and resources for video production. However, solo narration often relies heavily on auditory information, potentially limiting the amount of visual content. In contrast, other styles, like PPT or class, provide more comprehensive audio-visual information but are more common on long-video platforms. These formats, though information-rich, tend to be longer (often more than 10 min) and may not be ideally suited for the general public, targeting mainly medical professionals. It is challenging for video creators to find an ideal style to deliver high-quality medical information concisely and understandably.

### Video quality

The choice of tools is crucial in assessing video quality. Though PEMAT, VIQI, GQS, and mDISCERN are widely utilized [6, 12–15, 37, 38], our experience suggests certain limitations. The origin, contents, and limitations of these tools are shown in the Additional file 2 and the Additional file 3.

Short videos like TikTok, while more accessible and easier to understand, often contain less information, leading to a higher PEMAT-U, a lower PEMAT-A, a lower VIQI-2 (accuracy), VIQI-3 (video shooting aids), mDISCERN-4 (references), and mDISCERN-5 (uncertainty). Despite their brevity, TikTok videos scored well in mDISCERN-1 (clear target) and mDISCERN-3 (balance), attributing to its rigorous doctor certification. TikTok’s high user engagement contributed to its strong performance in VIQI-1 (flow) and helped balance its overall VIQI and GQS. Bilibili’s higher proportion of non-professional uploaders potentially affected its VIQI-2 (accuracy), mDISCERN-2 (reliability), and mDISCERN-3 (balance) scores. Table 5 also proves the inferior quality of videos uploaded by non-professionals. TikTok’s lowest VIQI-4 (appropriate title) score is due to its interface, where videos auto-play without user selection based on titles, unlike YouTube and Bilibili. Thus, titles are not very important on TikTok.

In general, videos on laryngeal cancer on YouTube are better than those on Bilibili and TikTok due to YouTube’s prioritized algorithms for health-related videos, the highest ratio of professionals especially non-profit organizations, and a more comprehensive range of video styles. These findings align with other comparative studies between YouTube and TikTok on different medical topics [39–41]. However, Bilibili and TikTok were found to have higher-quality gastric cancer videos than YouTube [5]. Additionally, the average quality of laryngeal cancer videos across all platforms was no better than videos about other diseases.

For Chinese audiences who cannot access YouTube, there is a recommendation for more translation (or secondary creation) of high-quality YouTube content for local platforms. Meanwhile, we also encourage translating high-quality Chinese videos into English and their subsequent upload to YouTube.

### Relationship between video quality and flow

Our study uncovered that the relationship between video quality and audience interaction was not strong. This observation, consistent with previous similar studies, suggests that viewers often cannot discern the quality of health-related videos [9, 32]. Public health education needs to be strengthened. Platforms should not rely solely on views and likes for video recommendations but rather enhance oversight and algorithm optimization to promote high-quality health content. This approach is crucial for ensuring viewers receive accurate and reliable health information.

### Limitations

The tools for video evaluation remain to be refined despite their wide application. Though we used four tools and three well-trained doctors rated the scores, we could not avoid the potential systemic biases. Secondly, certain data (such as video views on TikTok) are unavailable due to the platform restrictions. No platforms provide a thumb-down button to express opposition, so negative opinions remain unknown. Thirdly, these findings might not be fully applicable in different linguistic contexts. Fourthly, the additional text information (such as the textual introduction under the video page and the content of comments) was not included. Finally, this study’s findings represent a snapshot both in time and from a particular region in a limited sample size. With the rapid growth of social media platforms, the findings may vary greatly over time.

## Conclusions

This study provides reliable information for the public to understand the present state of laryngeal cancer-related online videos on social media platforms. The findings are helpful for the public, the content creators, and the platforms. Videos on social media platforms can help the public learn about the knowledge of laryngeal cancer to some extent. The short video platform, like TikTok, has strong interactive functions but carries less information. The long video platforms, like YouTube and Bilibili, have less flow but provide more information. In general, videos on YouTube are of the best quality but still need improvement. Chinese uploaders are encouraged to translate high-quality videos on YouTube and to post them on Chinese platforms. There is a call for more professional content creators to enhance the quality of videos related to laryngeal carcinoma, as some non-professionals might degrade the overall video quality. Video creators are facing the challenge of balancing the length and richness of content, endeavoring to deliver high-quality medical information concisely and understandably. They should also be aware of certification, originality, and the style of video shooting, which may help them make better videos and achieve more audience engagement. Finally, given the public’s limited ability to discern video quality, enhanced oversight and algorithm optimization for platforms are needed to promote high-quality health-related videos.

### Supplementary Information

Additional file 1. Details in Methods.

Additional file 2. Details in Assessment Tools.

Additional file 3. PEMAT (Patient Education Materials Assessment Tool).

## Data Availability

The data sets generated during and/or analyzed during this study are available from the corresponding author on reasonable request.
