# Supplementary material for: YouTube/ Bilibili/ TikTok videos as sources of medical information on laryngeal carcinoma: cross-sectional content analysis study
Source: BMC Public Health. 2024 Jun 14;24:1594. doi: 10.1186/s12889-024-19077-6 (PMC11177428; doi:10.1186/s12889-024-19077-6)
Supplement: Supplementary file 1 — Additional file 1. Details in Methods. [file 12889_2024_19077_MOESM1_ESM.docx]

**Details in Methods**

**1. Certification**

Meeting any one of the following conditions can be regarded as certification.

**1.1 YouTube**

①There is a blue label beneath the video showing this account belongs to a medical school/ hospital/ association/ doctor.

For example :


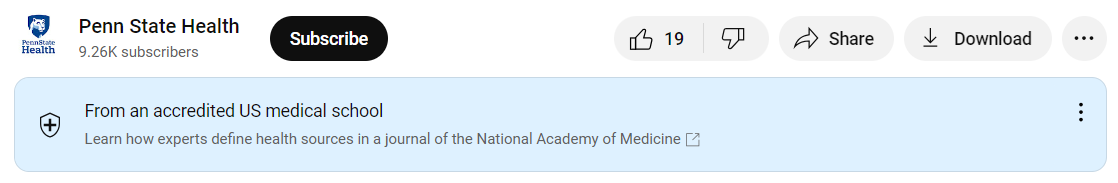


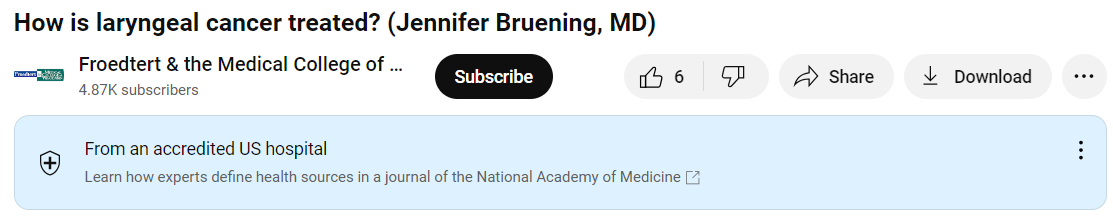


②There is a grey √ on the right of its ID.

For example :


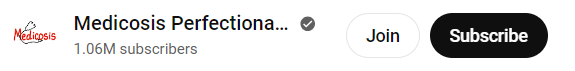


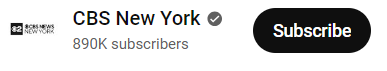


③ Some accounts get both the blue label and the grey √.

For example:


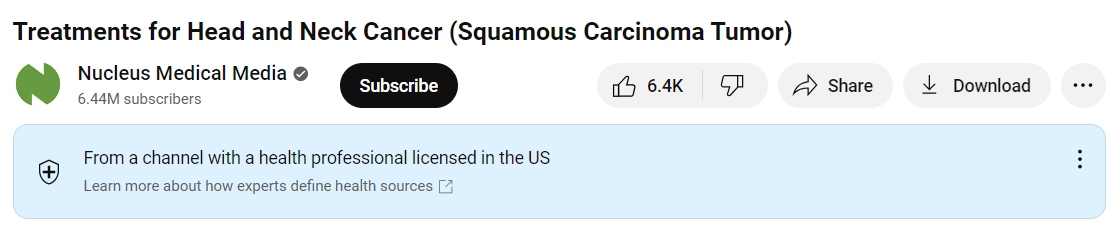


**1.2 Bilibili**

① Yellow flash: personal certification for a famous person/popular account.

For example:

There is a yellow flash on the lower right corner of its icon. This is a popular personal account on Bilibili with 165 thousand followers.


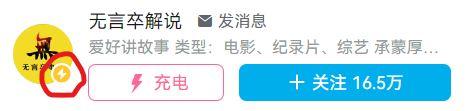


② Blue flash: certification for groups.

For example:

There is a blue flash on the right corner of its icon. This is an account from a hospital (Central Hospital of Zibo City, Shangdong Province, China) (hospital in Chinese: “医院”) with 1781 followers.


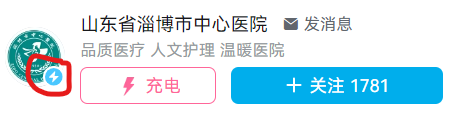


③ Grey V: personal occupational certification such as a doctor.

For example：

We can not see any yellow or blue flash on his icon. Then, we click on his homepage. We can see a grey V on the right of his homepage, which shows that he is a doctor. (doctor in Chinese: “医生” or“医师”)

On Bilibili, one who gets both a medical practitioner certificate and an employment certificate from any hospital can apply for a grey V.


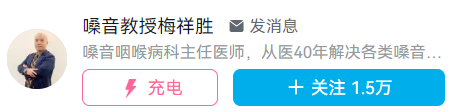


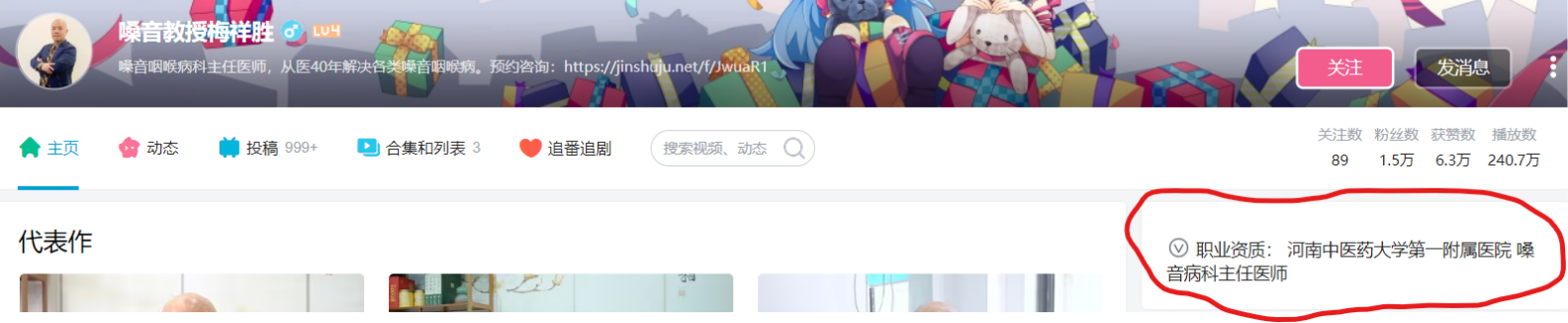


④ In theory, one account may get both the yellow flash and the grey V, but we could not find such an account in our study. On Bilibili, a doctor can apply for a grey V, but only when he gets enough followers (more than 50~100 thousand) can he apply for a yellow flash.

**1.3 TikTok**

1. Yellow V: personal certification for a famous person/popular account/ occupation.

On TikTok, only attending/associate/chief doctors who work in a grade 3 and first-class hospital (a hospital ranking system in China, grade 3 and first-class means the top level) can apply for a grey V. Resident doctors or any doctors who do not work in the grade 3 and first-class hospital can **NOT** apply it, and are NOT allowed to upload health-related videos on TikTok.

This strict rule of certification for doctors came into effect in June.2023.

For example:

There is a yellow V on the right of its ID. This is a chief doctor with 417000 followers.


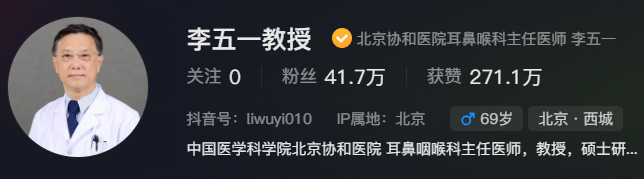


② Blue V: certification for groups.

For example:

There is a blue V on the right of its ID. This is an account from an official media, CCTV, with now 3072 thousand followers.


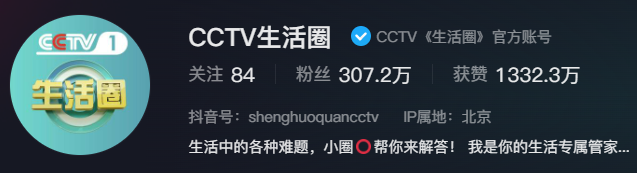


1. **Advertising**
   1. **YouTube**

On the YouTube browsing page, the label “sponsored” indicates that this is an ad (see the screenshot below). These ad videos have little relationship with users’ search term (They are just ads and this is how YouTube makes money.) and can not be enrolled in our study. Users can know it is an ad without clicking and watching it. That’s why we “skipped” it.


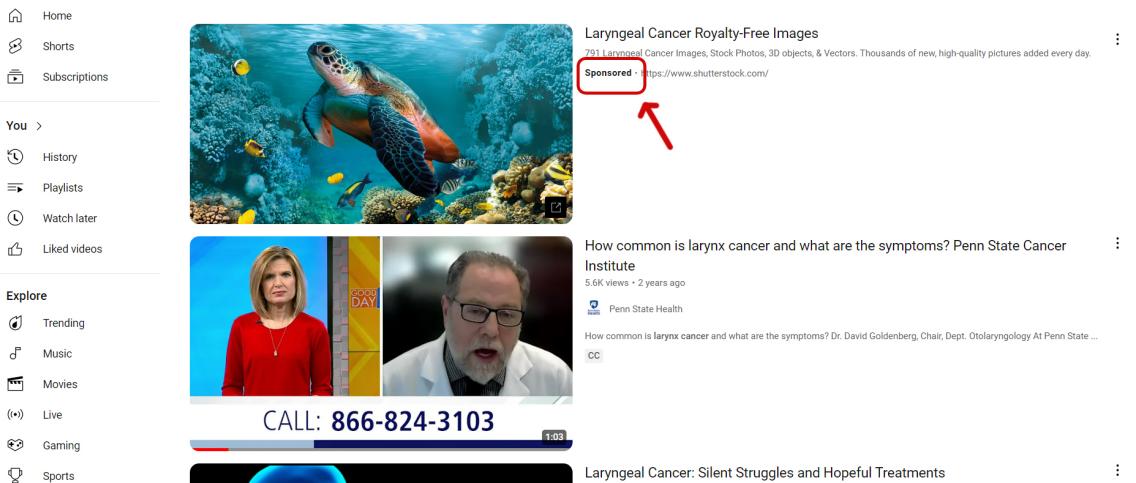


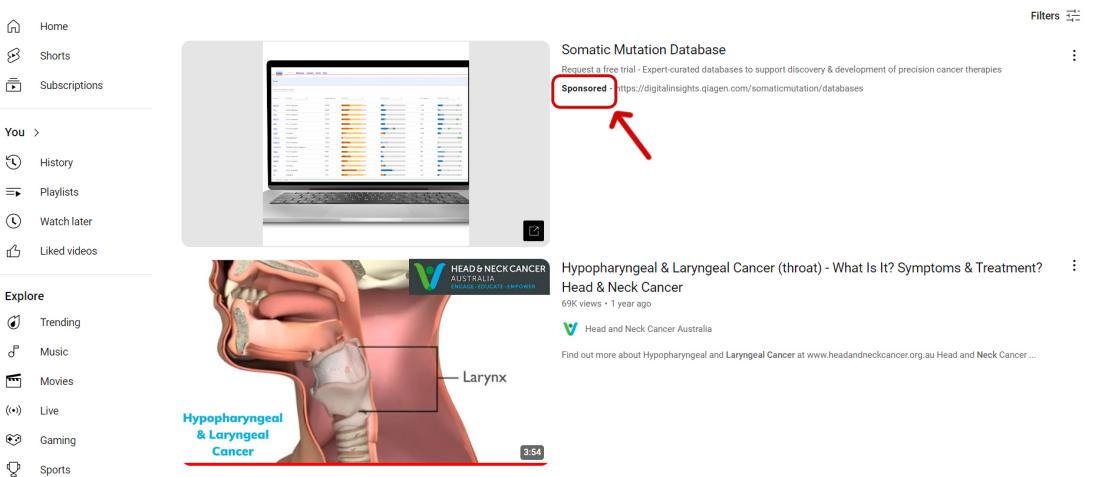


- 1. **Bilibili and TikTok**

Unlike YouTube, neither Bilibili nor TikTok has ad videos with “sponsored” labels.

- 1. **Other findings**

Our study did not consider a video as an ad if its primary objective was knowledge dissemination rather than profit generation (such as product demos). Unlike diabetes or hypertension, we can sell portable home-use glucometers or electronic sphygmomanometers. However, there is not much to sell for throat cancer patients. The number of these patients is less than that of patients with hypertension/diabetes/other common diseases. Therefore, throat cancer has very little commercial value. This could explain why we found few advertising videos in this field.

**3. Originality**

The following situation will be defined as non-originality:

①Others’ watermarks.

②Absence of the originality label.

*What is an “originality label”? This function on Bilibili is not available on YouTube and TikTok.

For example, The red arrow refers to the originality label. This is an original video uploaded by 四川卫视 (Sichuan Satellite TV).


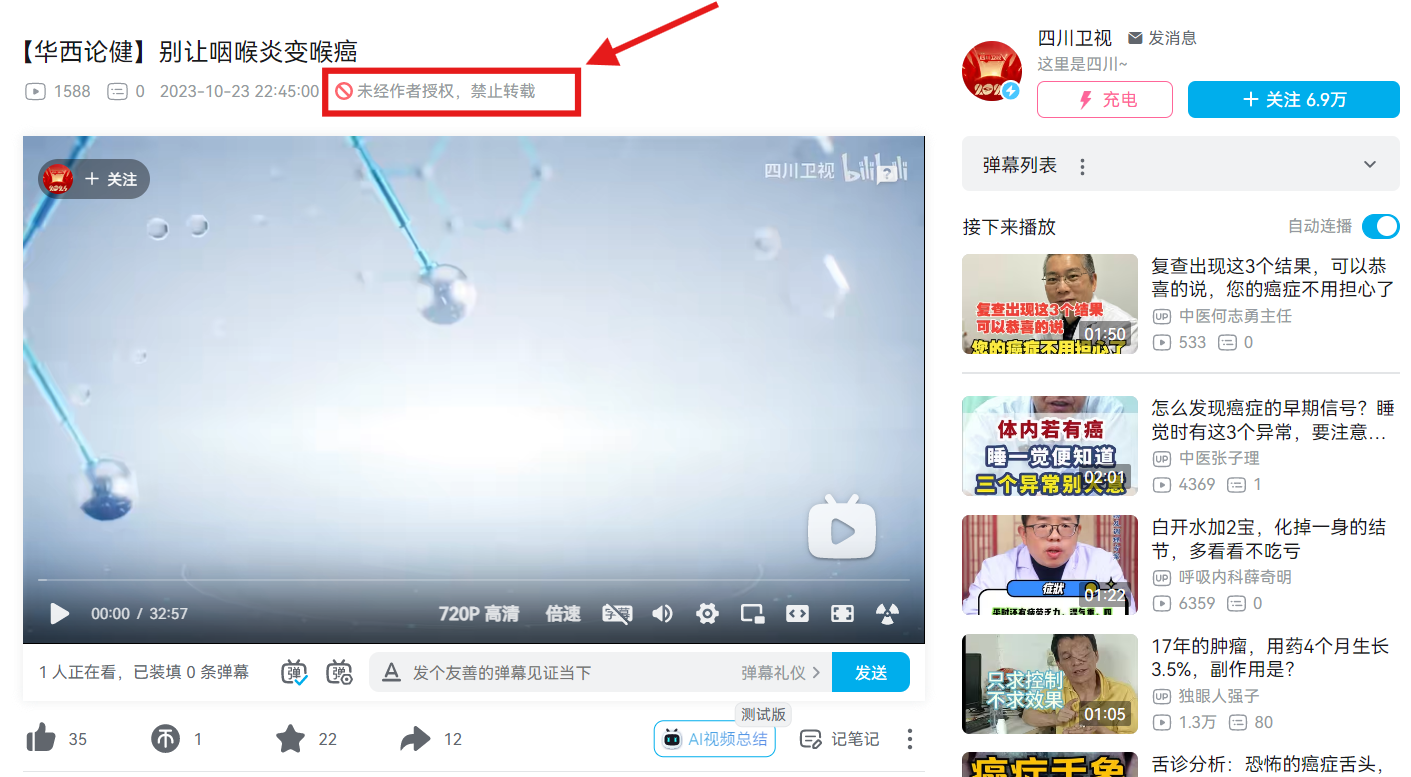


For another example, the red arrow refers to the absence of the originality label. The green arrow shows that the uploader’s ID is not consistent with the name in the video. This means that the video is not original but a repost.


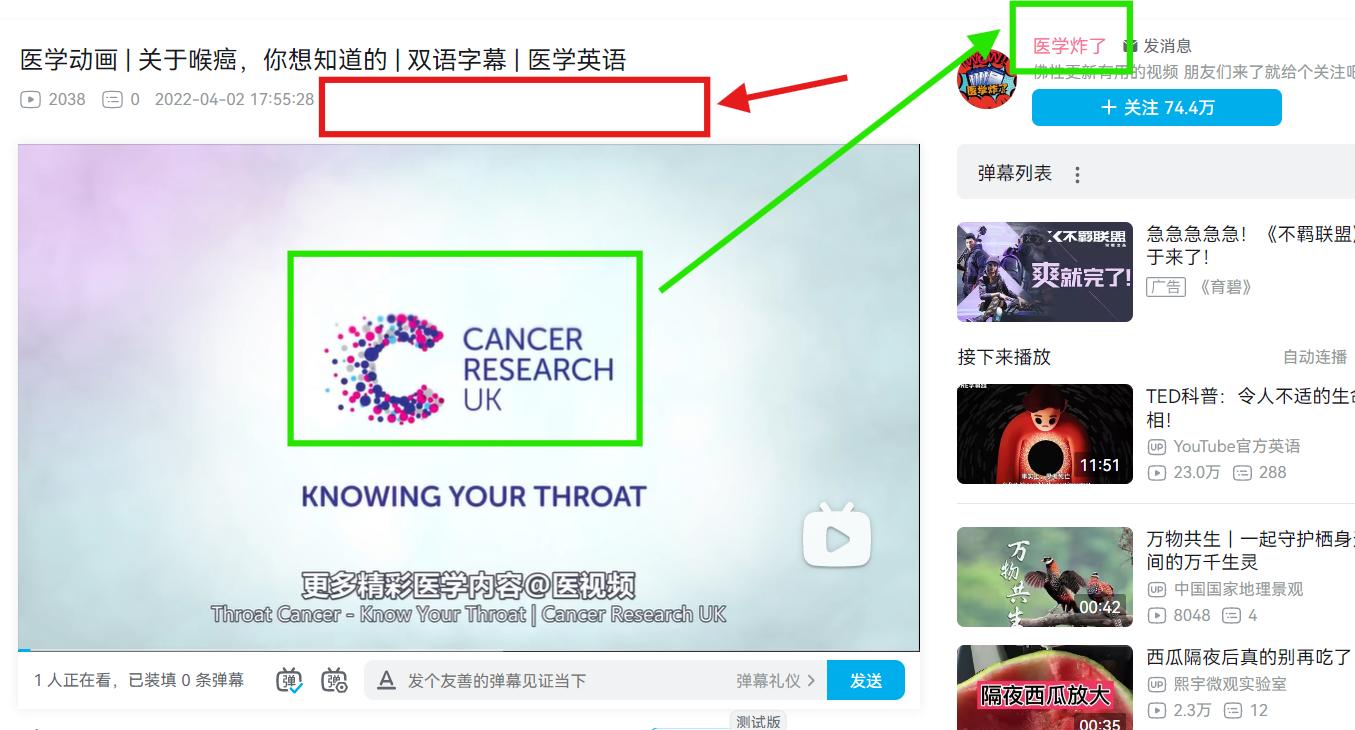


③ Repost, translation, and gross re-edition are not considered originality.

* We must acknowledge that the identifying originality is subjective, but we have tried our best to ensure each judgment is reasonable.

Advantages include allowing high-quality videos from other platforms to be shown on Bilibili, and facilitating knowledge dissemination. Disadvantages include the presence of similar videos (e.g. a documentary was edited into similar videos of varying lengths, and an English video on YouTube was translated into Chinese and uploaded by different people.), potentially detracting from user experience. Bilibili’s copyright policy requires refinement to balance these advantages and disadvantages.

**4. Inclusion and exclusion criteria**

**4.1 Inclusion criteria**

① Searching Date: January 1, 2024

② Searching keywords: YouTube “laryngeal carcinoma” and “throat cancer” in English; Bilibili and TikTok “喉癌” (laryngeal carcinoma in Chinese).

* “喉癌” is the same in both scientific name and common colloquial term and the same in both simplified Chinese and traditional Chinese characters.

③ Order: the default order without any filtering criteria.

④ No “sponsored” label (see 2.1).

⑤ Published over a week. (According to the services from the platforms, the data on views and likes are not stable during the first week and can not accurately reflect audience engagement.)

After the inclusion criteria, we had the top 100 videos from each platform (3×100 = 300 videos). However, we had not watched the whole video yet and did not know whether a video was duplicated or irrelevant. Then, we moved to the exclusion criteria part.

**4.2 Exclusion criteria**

① Similar videos. If two videos are similar (completely the same or edited from the same resource), the one uploaded by a certified account remains. If neither video was certified, the one uploaded first remains.

② Irrelevant videos. The topic of the videos was categorized as anatomy, etiology/ prevention, pathology, epidemiology, symptoms, examinations/ diagnosis, treatment, and prognosis. Videos not covering any of these topics were deemed irrelevant.

After the exclusion criteria, there were 248 videos left (see Figure 1).

**5. Style of video shooting**

① Solo narration: Only one person speaks in the video without other scenarios. Example：[www.youtube.com/watch?v=WLUQrzrx-Ao](http://www.youtube.com/watch?v=WLUQrzrx-Ao)

1. Questions and answers (Q&A): One person asks questions (or questions written in subtitles), and the other answers. Example: [www.youtube.com/watch?v=kChw5G3UJWU](http://www.youtube.com/watch?v=kChw5G3UJWU)
2. PPT/class: The videomaker uses PPT to show the video, such as some online medical classes for medical students. Example: [www.youtube.com/watch?v=TGttqUX4wQE&t=26s](http://www.youtube.com/watch?v=TGttqUX4wQE&t=26s)

④ Animation/action: Examples: [www.youtube.com/watch?v=TUetypm4gqQ](https://www.youtube.com/watch?v=TUetypm4gqQ)

[www.bilibili.com/video/BV1fu4y1o7xs/](http://www.bilibili.com/video/BV1fu4y1o7xs/)

⑤ Medical scenarios: The scenarios include but are not limited to when a patient is receiving treatment or consulting a doctor. Examples:  [www.youtube.com/watch?v=5kOCU_Ps-DI](https://www.youtube.com/watch?v=5kOCU_Ps-DI)

[www.douyin.com/video/7195753634271890749](http://www.douyin.com/video/7195753634271890749)

⑥ TV show/documentary: Example: <https://www.bilibili.com/video/BV1gY411j7ow/>

[www.youtube.com/watch?v=A6OmAHAnjjw](http://www.youtube.com/watch?v=A6OmAHAnjjw)

⑦ Others: Others that can not be clearly classified.
