# Supplementary material for: YouTube/ Bilibili/ TikTok videos as sources of medical information on laryngeal carcinoma: cross-sectional content analysis study
Source: BMC Public Health. 2024 Jun 14;24:1594. doi: 10.1186/s12889-024-19077-6 (PMC11177428; doi:10.1186/s12889-024-19077-6)
Supplement: Supplementary file 2 — Additional file 2. Details in Assessment Tools. [file 12889_2024_19077_MOESM2_ESM.docx]

**Details in Assessment Tools**

**1.mDISCERN** Modified DISCERN

The score of each question ranges from 0 to 1. The total score is 0 to 5.

mDISCERN-1 Are the aims clear and achieved?

0-no 1-yes

mDISCERN-2 Are reliable sources of information used?

0-no 1-yes

mDISCERN-3 Is the information presented balanced and unbiased?

0-no 1-yes

mDISCERN-4 Are additional sources of information listed for patient reference?

0-no 1-yes

mDISCERN-5 Are areas of uncertainty mentioned?

1. no 1-yes

Charnock originated the DISCERN tool in 1999 for evaluating textual health information[1]. However, this tool is not applicable to evaluating the information quality of video material[2]. In 2012, Singh.AG adapted Charnock’s DISCERN tool, and created the modified DISCERN tool to evaluate the reliability of video materials[3]. Since then, the modified version has been widely used (see the screenshot of the Pubmed search page below).


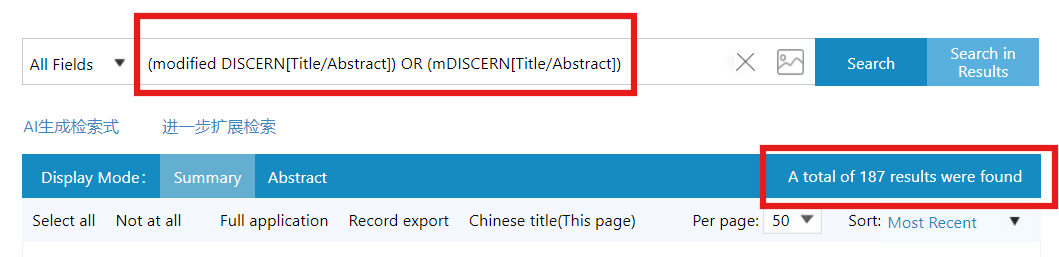


Advantage: ① Quick. ② Wide use.

Disadvantage: ① Readability and flow are not included. ② Limited questions. ③ Limited choice (only offering “no” or “yes” ).

**2. GQS** Global Quality Score

The total score is 1 to 5.

【Score 1】Poor quality, poor flow of the site, most information missing, not at all useful for patients.

【Score 2】Generally poor quality and poor flow, some information listed but many important topics missing, of very limited use to patients

【Score 3】Moderate quality, suboptimal flow, some important information is adequately discussed but others poorly discussed, somewhat useful for patients

【Score 4】Good quality and generally good flow, most of the relevant information is listed, but some topics not covered, useful for patients

【Score 5】Excellent quality and excellent flow, very useful for patients

Bernard invented the GQS tool in 2007 to rate the overall quality of a video[2]. Apart from overall quality, it takes into account the flow. Of all the tools, this is the simplest. Since then, the GQS tool have been widely used (see the screenshot of the Pubmed searching page below, the most used tool).


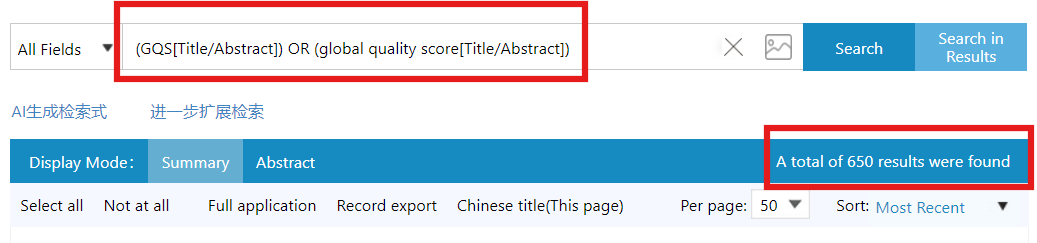


Advantage: ① Quick. ② Widest use.

Disadvantage: ① Limited questions. ② Not quantified clearly.

**3. VIQI** Video Information and Quality Index.

The score of each question ranges from 1 to 5. The total score is 5 to 20.

【VIQI 1】 information flow

*The VIQI 1 in our study is according to thumbs-up because the data of thumbs-up are available from all three platforms.

1 ＜10 thumbs-up

2 ＜100 thumbs-up

3 ＜1000 thumbs-up

4 ＜10000 thumbs-up

5 ≥10000 thumbs-up

* If a video turns off the bottom of thumbs-up (very few would do this), we rate its flow by views.

1 ＜100 views

2 ＜1000 views

3 ＜10000 views

4 ＜100000 views

5 ≥100000 views

【VIQI 2】 information accuracy

【VIQI 3】quality (videos including one point for each image, animation, interview, video captions, and summary)

【VIQI 4】 precision (level of coherence between video title and content)

In 2015, Nagpal addressed each component of the Global Quality Scale (GQS) and created the VIQI scale[5]. Each question is a 5-point Likert scale, but he did not clearly define “information flow”. Thus, we quantified it as the number of thumbs-up (because the data of thumbs-up are available from all the three platforms).

Although the VIQI scale is used by fewer people than other scales (see the screenshot of the Pubmed search page below), we regard it a supplement to the GQS scale.


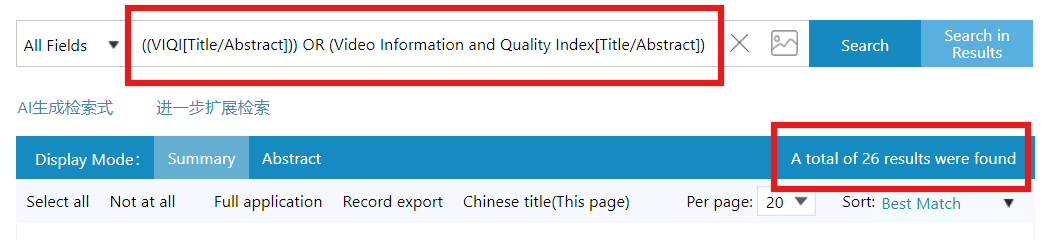


Advantage: ① Quick. ② Supplement for GQS.

Disadvantage: ① Limited questions. ② Not quantified clearly (we have quantified VIQI-1, but VIQI-2 and VIQI-4 can not be quantified). ③ Not wide use.

**4. PEMAT** Patient education materials assessment tool

Because of the complexity of this tool, it can not be shown in the Word, more details shown in the Excel (Additional file 3, or http://www.ahrq.gov/pemat ).

The PEMAT tool is the most comprehensive tool among the four tools in our study. Shoemaker created this tool in 2014 and confirmed its strong internal consistency, reliability, and evidence of construct validity[6]. Further study also confirmed its moderate to excellent interrater reliability[7]. Since then, the PEMAT tool has been widely used (see the screenshot of the Pubmed search page below).


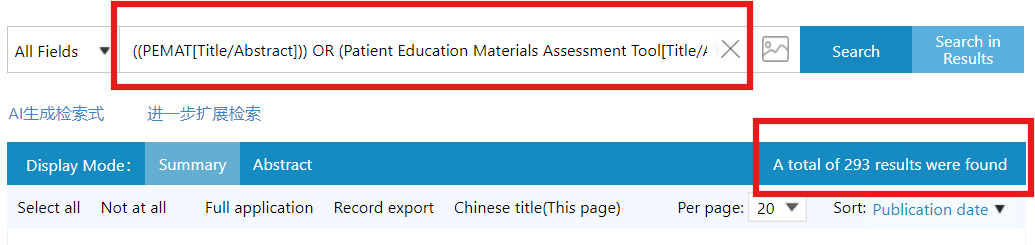


Advantage: ① Most detailed tool. ② Wide use. ③ Reliability and validity confirmed.

Disadvantage: ① Complex and time-consuming. ② “NA (non-applicable)” items potentially resulting in inflating score.

**References:**

1. Charnock D, et al. DISCERN: an instrument for judging the quality of written consumer health information on treatment choices. J Epidemiol Commun H. 1999; 53(2):105-111.

2. Azer SA. Are DISCERN and JAMA Suitable Instruments for Assessing YouTube Videos on Thyroid Cancer? Methodological Concerns. J Cancer Educ. 2020; 35(6):1267-1277.

3. Singh AG, Singh S, Singh PP. YouTube for information on rheumatoid arthritis--a wakeup call? J Rheumatol. 2012; 39(5):899-903.

4. Bernard A, et al. A systematic review of patient inflammatory bowel disease information resources on the World Wide Web. Am J Gastroenterol. 2007; 102(9):2070-2077.

5. Nagpal SJ, et al. YouTube videos as a source of medical information during the Ebola hemorrhagic fever epidemic. Springerplus. 2015; 4:457.

6. Shoemaker SJ, Wolf MS, Brach C. Development of the Patient Education Materials Assessment Tool (PEMAT): a new measure of understandability and actionability for print and audiovisual patient information. Patient Educ Couns. 2014; 96(3):395-403.

7. Vishnevetsky J, Walters CB, Tan KS. Interrater reliability of the Patient Education Materials Assessment Tool (PEMAT). Patient Educ Couns. 2018; 101(3):490-496.
